# Antimicrobial Resistance and Virulence Genes in* Enterococcus faecium* and* Enterococcus faecalis* from Humans and Retail Red Meat

**DOI:** 10.1155/2019/2815279

**Published:** 2019-05-09

**Authors:** Majda Golob, Mateja Pate, Darja Kušar, Urška Dermota, Jana Avberšek, Bojan Papić, Irena Zdovc

**Affiliations:** ^1^Institute of Microbiology and Parasitology, Veterinary Faculty, University of Ljubljana, Gerbičeva 60, SI-1000 Ljubljana, Slovenia; ^2^National Laboratory of Health, Environment and Food, Gosposvetska ulica 12, SI-4000 Kranj, Slovenia

## Abstract

The emergence of antimicrobial-resistant and virulent enterococci is a major public health concern. While enterococci are commonly found in food of animal origin, the knowledge on their zoonotic potential is limited. The aim of this study was to determine and compare the antimicrobial susceptibility and virulence traits of* Enterococcus faecalis* and* Enterococcus faecium* isolates from human clinical specimens and retail red meat in Slovenia. A total of 242 isolates were investigated: 101 from humans (71* E. faecalis*, 30* E. faecium*) and 141 from fresh beef and pork (120* E. faecalis*, 21* E. faecium*). The susceptibility to 12 antimicrobials was tested using a broth microdilution method, and the presence of seven common virulence genes was investigated using PCR. In both species, the distribution of several resistance phenotypes and virulence genes was disparate for isolates of different origin. All isolates were susceptible to daptomycin, linezolid, teicoplanin, and vancomycin. In both species, the susceptibility to antimicrobials was strongly associated with a food origin and the multidrug resistance, observed in 29.6% of* E. faecalis* and 73.3%* E. faecium *clinical isolates, with a clinical origin (Fisher's exact test). Among meat isolates, in total 66.0% of* E. faecalis *and* E. faecium* isolates were susceptible to all antimicrobials tested and 32.6% were resistant to either one or two antimicrobials. In* E. faecalis*, several virulence genes were significantly associated with a clinical origin; the most common (31.0%) gene pattern included all the tested genes except* hyl*. In meat isolates, the virulence genes were detected in* E. faecalis* only and the most common pattern included* ace*,* efaA*, and* gelE* (32.5%), of which* gelE* showed a statistically significant association with a clinical origin. These results emphasize the importance of* E. faecalis* in red meat as a reservoir of virulence genes involved in its persistence and human infections with reported severe outcomes.

## 1. Introduction

Enterococci are ubiquitous bacteria that primarily inhabit the intestinal tract of humans and warm-blooded animals, where they are part of the normal microbiota [1]. In addition, they are found in many foods of animal and plant origin as they are able to survive many adverse environmental conditions and play an important beneficial role in the production of various traditional fermented foods with unique organoleptic properties [1–4]. They are also employed in the biopreservation of foodstuffs as they produce several bactericidal substances like lactic acid and bacteriocins (enterocins) [5, 6]. The latter exert antimicrobial activity against several important Gram-positive foodborne pathogens including* Listeria monocytogenes*,* Staphylococcus aureus*, and* Clostridium botulinum* [7–12]. Enterococci are also used as probiotics for humans and animals, but their ability to acquire virulence and antibiotic resistance genes through horizontal gene transfer should be considered as a significant obstacle to their use as probiotics or as starter/adjunct cultures in foods [6, 8, 13].

Enterococci are among the leading nosocomial pathogens; they can be transmitted person-to-person, and also through contaminated food or environment, causing soft tissue or wound infections, bacteraemia, endocarditis, and especially infections of the urinary tract [3, 8, 14–16]. Due to their ability to invade the extraintestinal regions by translocation across an intact intestinal epithelium, they can shift from commensals to pathogens [17].* Enterococcus faecalis* and* Enterococcus faecium* are the most common enterococcal species detected in clinical and food samples. In the first wave of nosocomial enterococcal infections,* E. faecalis* was responsible for approximately 90% of the human infections and* E. faecium* for the remaining 10% [1, 18, 19]. However, over the past two decades, the second wave has commenced with* E. faecium*, which is much more frequently resistant to vancomycin (VRE), ampicillin (ARE), and high levels of aminoglycosides (HLAR) than* E. faecalis* [20, 21].

Enterococci are intrinsically resistant or tolerant to many antimicrobials and easily acquire the high-level drug resistance via horizontal gene transfer. The natural resistance of* E. faecalis* and* E. faecium* includes cephalosporins, aminoglycosides (low-level resistance), macrolides, and sulphonamides, also clindamycin and quinupristin/dalfopristin in* E. faecalis* [22]. Moreover, enterococci are showing the potential for resistance to virtually all antimicrobials used in human infections [23]. Some strains are multidrug-resistant (MDR), i.e., resistant to three or more groups of antimicrobial agents [1, 23, 24]. The resistance to vancomycin or teicoplanin is of special concern due to the important therapeutic use of these agents against the MDR enterococci and other Gram-positive bacteria [23, 24]. Ampicillin, vancomycin, and gentamicin are the most relevant antimicrobials for the treatment of MDR enterococcal infections, but the extensive use of vancomycin generated a raise in the number of VRE that constitute a serious risk group [4, 25]. Enterococci resistant to antimicrobials, including VRE, play an important role in the inter- and intraspecies transfer of antimicrobial resistance genes [26].

Since enterococci are present in the intestine of animals, contamination of meat during slaughter is common. Enterococci should be screened for specific genetic traits that determine their virulence potential, aiming also to confirm their zoonotic transmission, which represents a serious health concern [27, 28]. Many factors determine the virulence of* Enterococcus* species, for example, the ability to colonize the gastrointestinal tract or to adhere to a range of extracellular matrix proteins or to the epithelial cells [29]. Several enterococcal virulence genes that may be involved in the onset of a disease in humans or exacerbation of the disease symptoms have been described [30]. Importantly, many of these determinants are also found in the strains isolated from foods [3, 31]. The aggregation substance (*asa1*), gelatinase (*gelE*), cytolysin (*cylA*), enterococcal surface protein (*esp*), hyaluronidase (*hyl*), collagen‐binding‐protein (*ace*), endocarditis antigen (*efaA*), and extracellular superoxide are among the most important enterococcal virulence determinants [4, 32–34].

The aim of the present study was to determine and compare for the first time in Slovenia the antimicrobial resistance and the presence of virulence genes in* E. faecalis* and* E. faecium* isolates recovered from human patients in 2016–2018 and from fresh beef and pork in 2017.

## 2. Materials and Methods

### 2.1. Enterococci from Human Clinical Specimens

Enterococci were isolated in the National Laboratory of Health, Environment and Food, Slovenia. From January 2016 to June 2018, a total of 14* E. faecium* and 16* E. faecalis* isolates were retrieved from blood cultures. From March to June 2018, 16* E. faecium* isolates were obtained from other clinical specimens, i.e., urine (*n*=11), tracheal aspirate (*n*=2), abdominal drainage aspirate (*n*=1), central venous catheter (*n*=1), and wound (*n*=1). In the same period, 55* E. faecalis *isolates from urine (*n*=38), vagina (*n*=8), wound (*n*=4), primary sterile sites (*n*=3), ear (*n*=1), and ejaculate (*n*=1) were obtained. In total, 101 isolates (71* E. faecalis* and 30* E. faecium*) were retrieved from clinical samples collected from the patients during 2016–2018 in Slovenia. All specimens were cultivated in different selective and nonselective media according to the standard protocols [35]. The suspect colonies were identified by the matrix-assisted laser desorption/ionization time-of-flight (MALDI-TOF) mass spectrometry (Microflex LT system; Bruker Daltonics, Germany) according to the manufacturer's instructions.

### 2.2. Enterococci from Red Meat

From January to December 2017, 141* E. faecalis* and* E. faecium* isolates were retrieved from unpacked and packed chilled fresh pork and beef of Slovenian and foreign origin. A total of 70 isolates (60* E. faecalis*, 10* E. faecium*) were collected from fresh pork and 71 isolates (60* E. faecalis*, 11* E. faecium*) from fresh beef samples. Sampling was performed throughout the territory of Slovenia as a part of the national monitoring within the framework of the European baseline study on antimicrobial resistance. Sampling took place in the retail establishments that directly supply the final consumer (trade). Original packages of pre-packed meat were randomly selected from the sales display. In the butcher's shops, the sampler randomly selected a piece of meat in the total weight of at least 100 g. Fresh beef and pork samples were collected throughout the year and transported in the cooling boxes to the laboratory of the Veterinary Faculty, Slovenia. Twenty-five grams of each meat sample was supplemented with 225 ml of buffered peptone water (Biolife, Italy), homogenized in a stomacher, and incubated at 37°C for 16–20 h. Subsequently, the liquid enrichment culture was spread with a 10-*μ*l loop onto the selective Slanetz Bartley agar (Biolife, Italy) and ChromID VRE chromogenic selective agar (bioMerieux, France) for the detection and differentiation of* E*.* faecium* and* E. faecalis* showing acquired vancomycin resistance. The agar plates were incubated at 37°C for 24–48 h. Isolates with typical morphology were selected and pure subcultures from single colonies on the blood agar plates were obtained. The suspect colonies were identified by MALDI-TOF mass spectrometry (see above).

### 2.3. Antimicrobial Susceptibility Testing

Isolates were phenotypically tested for their susceptibility to 12 different antimicrobials using a broth microdilution method to determine the minimum inhibitory concentration (MIC). A total of 242 isolates were tested with a commercially available 96-well broth microdilution plate (EUVENC, Sensititre, Trek Diagnostic Systems; Thermo Scientific, USA) following the manufacturer's instructions and including the following antimicrobials: ampicillin (AMP), chloramphenicol (CHL), ciprofloxacin (CIP), daptomycin (DAP), erythromycin (ERY), gentamicin (GEN), linezolid (LZD), quinupristin/dalfopristin (Synercid, SYN), teicoplanin (TEI), tetracycline (TET), tigecycline (TGC), and vancomycin (VAN). MICs were determined after 24 h of incubation at 35°C in aerobic conditions using the Sensititre cation adjusted Mueller-Hinton broth with TES (CAMHBT, Sensititre, Trek Diagnostic Systems; Thermo Scientific, USA) according to the manufacturer's instructions. The MIC endpoint was determined as the next dilution above the last dilution where growth was observed. Reference strain* E. faecalis *ATCC 29212 was used as a control.

*E. faecalis* and* E. faecium* isolates were classified as susceptible or resistant based on the epidemiological cut-off values (ECOFFs) according to the European Committee on Antibiotic Susceptibility Testing (EUCAST) [36] and the recommendations of the EU Reference Laboratory for Antimicrobial Resistance (EURL-AR) [37]. The evaluation was based on the interpretation of MIC values obtained in concordance with the Decision 2013/652/EU of the European Commission. Because* E. faecalis* exhibited the intrinsic resistance to quinupristin/dalfopristin, MIC data for SYN were not included in Table S1 and Table S2. The intrinsic resistances were adopted from the EUCAST expert rules [38] and were excluded from the result tables except for gentamicin, to which enterococci exert only a low level of intrinsic resistance. MIC_50_ and MIC_90_ were also determined, both for human and meat isolates, describing MICs of the tested antimicrobials required to inhibit the growth of 50% and 90% of the obtained* E. faecalis* or* E. faecium* isolates, respectively (Table S1 and Table S2).

### 2.4. Molecular Detection of Virulence Factors

Genes encoding the enterococcal virulence factors* ace*,* asa1*,* cylA*,* efaA*,* esp*,* gelE*, and* hyl* were detected using PCR. DNA was extracted from the bacterial cultures grown on the sheep blood agar plates with a simple cell lysis (boiling at 95°C for 15 min, centrifugation at 14,000×*g* for 2 min). The supernatant was used as a template for PCR without further purification. Virulence genes were detected using two multiplex PCR tests: PCR 1 for the detection of* asa1*,* cylA*,* esp*,* gelE*, and* hyl* [32] and PCR 2 for the detection of* ace *and* efaA* [39]. Briefly, a 25-*μ*l reaction mixture for both PCR assays contained 12.5 *μ*l of 2× Multiplex PCR Master Mix (Qiagen, Germany), 2.5 *μ*l of 10× primer mix (containing 2 *μ*M of each primer for* asa1*,* gelE*, and* hyl*; 1 *μ*M of each primer for* cylA* and* esp* for PCR 1; and 2 *μ*M of each primer for* ace* and* efaA* for PCR 2), and 2.5 *μ*l of DNA template. An initial activation step at 95°C for 15 min was followed by 30 cycles of denaturation at 94°C for 30 sec, annealing for 90 sec (PCR 1: 56°C, PCR 2: 55°C), and extension at 72°C (PCR 1: 60 sec, PCR 2: 90 sec), followed by one cycle at 72°C for 10 min. Amplicons were detected using the QIAxcel capillary electrophoresis system (Qiagen, Germany).

### 2.5. Statistical Analysis

The Fisher's exact test implemented in the GraphPad Prism v6.01 (GraphPad Software, USA) was used to assess the association between different traits (origin of isolation, virulence gene, resistance phenotype). To compare the isolates according to the origin of isolation, isolates from beef and pork samples were joined into a single group and compared to human isolates. To assess the association between the antimicrobial resistance phenotypes and virulence genes, isolates of different origin were joined into a single group and compared according to the resistance pattern. Each species was analyzed independently and only groups with an expected frequency of >5 were compared. For the analysis of correlation between the antimicrobial resistance and virulence, the number of cooccurring resistant phenotypes and virulence genes was considered. The* p* value of ≤0.05 was considered as statistically significant.

## 3. Results and Discussion

Several studies demonstrated* E. faecalis* and/or* E. faecium* to be the most common enterococcal species found in food of animal origin [40–44]. This is in accordance with the present study, as* E. faecalis *and* E. faecium* were the predominant species isolated from red meat, found in 69.5% and 11.3% of the samples, respectively. In addition,* Enterococcus* species* E. hirae*,* E. casseliflavus*,* E. durans, E. devriesei*,* E. gilvus, E. mundtii*, and* E. thailandicus* were also isolated from beef and pork samples (data not shown).* E. faecium* and* E. faecalis* were also the predominant enterococcal species isolated from healthy cattle, pigs, and chicken in nine EU countries, detected in 30.6% and 25.7% of the investigated samples, respectively [45]. The presence of enterococci in food is considered as an indicator of faecal or environmental contamination and represents a potential risk to human health [26]. Enterococcal endocarditis remains one of the most difficult enterococcal infections to treat due to the high level of antimicrobial resistance observed [35, 46]. Enterococcal bacteraemia, associated with high mortality rates [35, 46], represented 5.4–8.1% of human bloodstream infections in 2006–2011 in Slovenia [47].

### 3.1. Antimicrobial Resistance

**Figure 1:**
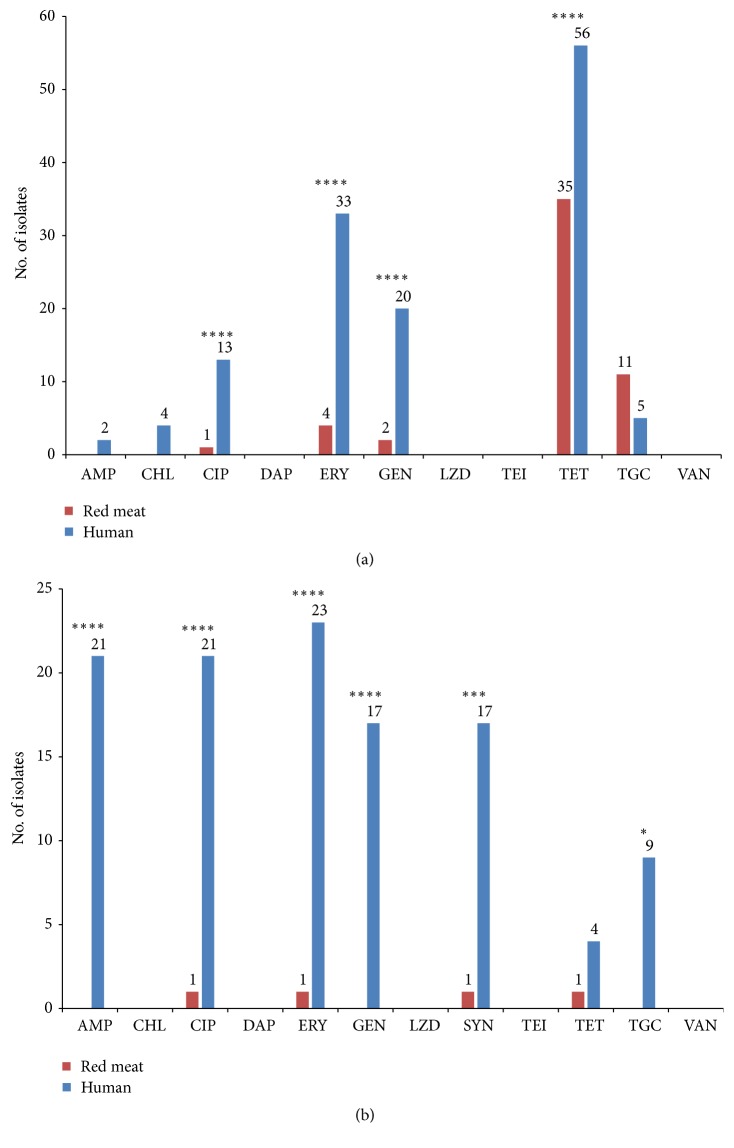
Antimicrobial resistance in* Enterococcus* isolates from human clinical specimens and red meat. (a)* Enterococcus faecalis* isolates (*n* = 191). (b)* Enterococcus faecium *isolates (*n* = 51). Numbers at the top of each column indicate the number of isolates; only numbers ≥1 are shown. Significant associations (the Fisher's exact test) of antimicrobial resistance and origin of isolation for each species are indicated with asterisks. Significance levels: ^*∗*^*p*<0.05; ^*∗∗∗*^*p*<0.0005; ^*∗∗∗∗*^*p*<0.0001.

The antimicrobial susceptibility of* E. faecalis* and* E. faecium* isolates from humans and red meat differed significantly for both species, as shown in Table 1, Table S1, and Table S2. In both species, susceptible isolates were strongly associated with a food origin (*p*<0.0001); 65.9% of meat isolates were classified as susceptible compared to only 11.9% of human clinical isolates. All* E. faecalis* and* E. faecium* isolates from both origins were susceptible to daptomycin, linezolid, teicoplanin, and vancomycin. The MDR phenotype was significantly overrepresented among the human clinical isolates in comparison with the meat isolates for both species (*p*<0.0001; Table 1). Among the clinical isolates, mostly from blood cultures, 30.5% showed the MDR phenotype. Almost one-third (29.6%) of* E. faecalis* isolates from different clinical samples (blood cultures, urine, wound, vagina, and ejaculate) were classified as MDR and the most common resistance pattern was ERY-GEN-TET. In* E. faecalis*, a significant association with a clinical origin was identified for the following antibiotics: CIP (*p*<0.0001), ERY (*p*<0.0001), GEN (*p*<0.0001), and TET (*p*<0.0001) (Figure 1(a)). Furthermore, 73.3% of clinical* E. faecium *isolates from blood cultures, urine, and tracheal aspirate were MDR with the most common resistance pattern AMP-CIP-ERY-GEN-TGC. In* E. faecium*, a significant association with a clinical origin was identified for the following antibiotics: AMP (*p*=0.0002), CIP (*p*<0.0001), ERY (*p*<0.0001), GEN (*p*<0.0001), SYN (*p*=0.0001), and TGC (*p*<0.0001) (Figure 1(b)). Among the meat isolates, only two (1.7%)* E. faecalis* isolates showed the MDR resistance pattern (ERY-GEN-TET). Distributions of MICs, MIC50, and MIC90 for the human clinical and meat isolates are shown in Table S1 and Table S2.

In 2017, the Slovenian National Antimicrobial Susceptibility Testing Committee reported a low resistance of human clinical* E. faecalis* isolates to ampicillin (0.6%), linezolid (0.4%), vancomycin (0.04%), and ciprofloxacin (0.5%) [48]. A higher resistance of* E. faecalis* was observed for nitrofurantoin (24.2%) and high level of gentamicin (19.2%) [48].* E. faecalis *has acquired resistance to gentamicin, but the resistance to ampicillin and vancomycin is less common than in* E. faecium* [49]. In the present study, only 12.7% of clinical* E. faecalis* isolates from different clinical specimens were susceptible to all antimicrobials tested (Table 1). The majority of clinical* E. faecalis* isolates were resistant to tetracycline (78.9%), followed by the resistance to erythromycin (46.5%) (Table S1, Figure 1(a)). Twenty (28.2%) clinical* E. faecalis* isolates, originating from blood cultures, urine, vagina, operation wound, and ejaculate, showed the HLAR phenotype (Table S1); of these, 15 isolates also showed the resistance to tetracycline and erythromycin. In addition, two HLAR isolates from blood cultures were also resistant to ampicillin (MIC>64 *μ*g/ml) and ciprofloxacin (MIC>16 *μ*g/ml). A lower frequency of resistance in clinical* E. faecalis* isolates was noticed for ciprofloxacin (18.3%), tigecycline (7.0%), chloramphenicol (5.6%), and ampicillin (2.8%) (Table S1). These findings contrast a study which reported higher resistance rates of* E. faecalis* to tetracycline (88.0%), erythromycin (62.3%), and ciprofloxacin (39.4%) [50].

As for human clinical* E. faecium *isolates, low resistance was observed in Slovenia in 2017 for vancomycin (0.6%) and linezolid (0.5%), while higher resistance rates were reported for ampicillin (89.9%), ciprofloxacin (94.3%), and high level of gentamicin (49.1%) [48]. In the present study, most of the clinical* E. faecium* isolates were resistant to erythromycin (76.7%), ampicillin (70.0%), and ciprofloxacin (70.0%) (Table S1, Figure 1(b)). More than half (56.7%) of the isolates, originating from blood cultures, urine, and tracheal aspirate, were highly resistant to gentamicin (HLAR* E. faecium*) (Table S1); among these, 15 were resistant also to ampicillin, ciprofloxacin, and erythromycin. Resistance to quinupristin/dalfopristin was observed in 56.7% of clinical* E. faecium* isolates. Previous studies reported a wide range (1–70%) of quinupristin/dalfopristin resistance in human isolates [50, 51]. Furthermore, 30.0% of isolates were also resistant to tigecycline, while the proportion of tetracycline-resistant isolates was lower (13.3%). A higher rate of ampicillin resistance in* E. faecium* compared to* E. faecalis* isolates from human clinical samples is in congruence with previous studies reporting that* E. faecium* isolates acquire ampicillin and vancomycin resistance more frequently than* E. faecalis*, which is less efficient in accumulating resistance, although* E. faecalis* is responsible for more human infections than* E. faecium* [25, 52]. On the other hand,* E. faecium* is an important nosocomial pathogen and has acquired resistance to different classes of antimicrobials. Moreover, MDR* E. faecium* is associated with an increased mortality rate in humans [49, 53].

Based on the ECOFFs, all red meat isolates were susceptible to ampicillin, chloramphenicol, daptomycin, linezolid, teicoplanin, and vancomycin (Table S2 and Figure 1). Susceptibility to the last four antimicrobials is of particular importance since they are categorized as critically important antimicrobials (CIA) in human medicine. In the present study, VRE were not isolated from fresh pork and beef samples. This is in agreement with some studies reporting the absence of teicoplanin and vancomycin resistance in enterococci from red meat [41–43, 54], but in contrast with other studies describing the presence of VRE in raw meat [55, 56]. Almost one-third (32.6%) of red meat isolates were resistant to either one or two antimicrobials; two* E. faecalis* meat isolates were resistant to three (Table 1). In comparison, 63.0% of isolates resistant to at least one antimicrobial tested were reported in Turkey [44] and only 3.4% of enterococci from retail meat in Canada were susceptible to all antimicrobials tested [42]. In the present study,* E. faecalis* meat isolates were most often resistant to tetracycline (29.2%) (Table S2, Figure 1(a)). Reduced susceptibility among* E. faecalis* isolates was observed for tigecycline (9.2%) and ciprofloxacin (0.8%). Two isolates (1.7%), one from pork and one from beef, were classified as HLAR with MIC value for gentamicin >1024 *μ*g/ml.* E. faecium* meat isolates showed a very low frequency of antimicrobial resistance to ciprofloxacin, erythromycin, quinupristin/dalfopristin, and tetracycline (4.8% each) (Table S2, Figure 1(a)). Results of the present study were in congruence with previous studies on the resistance of enterococci in meat, with the exception of higher resistance to tetracyclines in previous reports [41, 42, 54].

In 2013, Slovenia and three other EU countries reported the antimicrobial resistance of enterococcal isolates from broiler, pig, and bovine meat [57]. Overall,* E. faecalis* isolates showed resistance to tetracyclines (42.2%), streptomycin (11.1%), and erythromycin (6.7%).* E. faecium* isolates were resistant to quinupristin/dalfopristin (50.0%) and tetracyclines (9.1%). In general, enterococci from pork and beef were less resistant in comparison with enterococci from broiler meat, except for chloramphenicol and linezolid. Slovenia reported resistance to the selected antimicrobials for 93* Enterococcus* isolates from broiler meat and for 52* E. faecalis* isolates from pork. Among the latter, 50.0% were resistant to tetracyclines, 21.2% to erythromycin, and 17.3% to streptomycin [57]. Interestingly, a higher rate of antimicrobial resistance in pork isolates was observed in 2013 than in the present study. According to the current EU legislation, the monitoring of antimicrobial resistance of enterococci (*E. faecalis* and* E. faecium*) from animals and derived meat is not mandatory. However, the surveillance of antimicrobial resistance in enterococci from meat, in particular if it is eaten raw and does not undergo the processing steps to eliminate live bacteria before consumption, is important for the assessment of possible zoonotic risks [58]. In the present study, the reduced susceptibility was also observed for some* E. hirae* and* E. durans* isolates from red meat with MIC value of 128 *μ*g/ml for tetracycline (data not shown), which may indicate a possible interspecies transfer of resistance determinants. Enterococci are considered as reservoirs of antimicrobial resistance genes, which can be transferred to humans via the food chain. Identification of resistance genes, in addition to the phenotypic characterization of resistance, may provide additional information for the studied isolates. However, according to the whole-genome sequencing (WGS),* E. faecalis* and* E. faecium* resistance genotypes correlated with the resistance phenotypes in 96.5% of cases for the 11 investigated antimicrobials [59]. This suggests that the phenotypic susceptibility testing cannot yet be fully replaced by WGS.

### 3.2. Virulence Genes

The results on the presence of virulence genes are summarized in Tables 2 and 3. As expected and in congruence with previous studies [31, 60–62], the virulence traits were more commonly detected in clinical than in meat isolates. A significant association between the presence of a virulence gene and a clinical origin was identified among* E. faecalis* isolates for* asa1* (*p*<0.0001),* cylA* (*p*<0.0001),* esp* (*p*<0.0001), and* gelE* (*p*=0.0005) genes (Table 2). This is in congruence with previous studies, reporting the enterococcal surface protein (*esp*) as one of the most important factors for colonization and persistence of* E. faecalis* in human urinary tract infections [63] and biofilm production [64]. The production of gelatinase (*gelE*) was also confirmed previously in clinical* E. faecalis* strains, but its connection with biofilm formation remains unclear [64]. A higher frequency of adhesion genes (*esp* and* asa1*) and gelatinase (*gelE*) was described for clinical* E. faecalis* isolates in comparison with* E. faecium* [65].

In the present study, none of the isolates harbored all of the virulence genes simultaneously. All the tested virulence genes were found in human clinical isolates, whereas in the red meat isolates all but* hyl* were detected (Table 2). A simultaneous presence of more than two virulence genes was demonstrated in 94.4% of* E. faecalis* and 13.3% of* E. faecium* isolates from humans and in 68.3% of* E. faecalis* isolates from red meat (Table 3). In the clinical* E. faecalis* isolates, 14 virulence gene patterns were discovered, with* ace-asa1-cylA-efaA-esp-gelE* being the most common as it was detected in one-third (*n*=22) of the isolates (Table 3), mostly from the urine samples (*n*=13) but also from the blood culture (*n*=3), vagina (*n*=3), wound (*n*=2), and ejaculate (*n*=1). Moreover, 73.2% (*n*=52) of clinical* E. faecalis* isolates harbored four or more virulence genes at the same time, compared to only 10.0% (*n*=2) of clinical* E. faecium* isolates. These findings contrast with a study which reported that isolates linked with bacteraemia did not show any particular propensity for the carriage of virulence genes, whereas isolates from the urinary tract infections usually possessed two to four virulence traits [66]. Among the six distinct virulence gene patterns found in clinical* E. faecium *isolates,* esp-hyl* was the most frequent as it was detected in half (*n*=16) of the isolates (Table 3), mostly from the blood cultures (*n*=10) but also from the urine samples (*n*=4) and tracheal aspirates (*n*=2). Moreover, far less clinical* E. faecium* isolates harbored virulence genes than* E. faecalis*, with the exception of* esp* (Table 3). The* hyl* gene was found in 53.3% of* E. faecium* and in only 2.8% of* E. faecalis* clinical isolates (Table 2). This gene is widely distributed among the clinical* E. faecium* isolates and it was previously considered as restricted to this species [67]. However, it has been recently described also in* E. faecalis *[28, 65, 68], which supports the findings of the present study. In addition, herein we showed that* esp* and* hyl* were overrepresented among* E. faecium* isolates of a clinical origin in comparison with food isolates (*p*<0.0001) (Table 2). The incidence of* esp *in clinical* E. faecium *is reported to be increasing compared to clinical* E. faecalis *isolates [50]. The carriage of* esp*, coding for the enterococcal surface protein, in enterococci from foods and humans has also been described before [31, 69]. However, it was more frequently observed in clinical isolates than in commensal isolates [70] and it was found in a low proportion in the meat samples [71, 72]. In the present study,* esp* was detected in equally high proportions of clinical* E. faecalis* and* E. faecium*, while its occurrence in the red meat* E. faecalis* isolates was much lower.

Genes coding for the virulence were not detected in 26.6% of clinical* E. faecium* isolates nor in any of* E. faecium* isolated from meat (Table 3). However, according to the literature, the presence of* asa1*,* cylA*,* efaA*,* esp*,* gelE*, and* hyl* genes has been confirmed in* E. faecium* isolates from meat and meat products [71, 73]. In general,* E. faecalis* isolated from food is showing more virulence traits than* E. faecium* [71].* E. faecium* of animal origin is not highly important for human infections, but should be considered in the view of transferring the resistance genes to other pathogenic enterococci [74]. Furthermore, also* E. hirae* and* E. mundtii* could represent a reservoir of virulence genes, as in the present study* ace*,* asa1*, and* efaA* were found in* E. hirae* and* ace*,* efaA*, and* gelE *in* E. mundtii* (data not shown). On the other hand,* E. faecalis* of animal origin was reported as a human hazard* per se* as the same types of* E. faecalis* were found in animals, meat, human faecal samples, and patients with enterococcal bacteraemia [74].

Results of the present study showed a high prevalence of virulence genes in* E. faecalis *isolates from meat (Table 2). More than three virulence genes were detected in 68.3% of isolates (Table 3), which is a much higher percentage than previously reported [62]. Furthermore, 97.5% of isolates harbored at least one virulence gene (Table 3). This is in accordance with a previous study, in which a higher percentage of* E. faecalis* isolated from four meat types, including beef and pork, was reported [42]. In the present study, the* ace-efaA-gelE* virulence gene pattern was the most common for* E. faecalis* from meat. This is an important finding as* efaA* and* gelE* were shown to be associated with the exacerbation of infective endocarditis in humans [75, 76]. In addition, it has been shown before that the presence of* ace* is often confirmed in clinical and retail meat* E. faecalis* isolates [42, 66, 71]. This is in accordance with the results of the present study, showing that three-quarters of* E. faecalis* isolates from both origins harbored* ace* while it was found in only few clinical* E. faecium *isolates (Table 2).

Similarly, the concurrent expression of virulence factors cytolysin and aggregation substance was reported to result in the increased pathogenicity of* E. faecalis* isolates [77]; herein, the presence of both* cylA* and* asa1* genes was confirmed in a small number of* E. faecalis* isolates from red meat. Regardless of the gene patterns, the presence of* asa1* was demonstrated in one-third of* E. faecalis* meat isolates (Table 2), but its frequency in* E. faecalis* food isolates was previously reported to be high [31, 42, 60, 71]. In the present study,* cylA* was confirmed in low numbers of* E. faecalis* meat isolates and* E. faecium* clinical isolates, while it was more abundant in clinical* E. faecalis* (Table 2). Previously, this gene was also reported in low numbers of isolates originating from fermented dry sausages, beef, and pork [42, 71].

Gene encoding the endocarditis antigen was the most frequently detected virulence gene in both clinical and meat* E. faecalis *isolates in the present study (Table 2), which is in congruence with a study hypothesizing that* efaA* is important also for the persistence of enterococci in environments other than human tissues [78]. On the other hand,* efaA* was only found in few clinical and none of the meat* E. faecium* isolates (Table 2), which contrasts with the study reporting 63%* efaA-*positive* E. faecium *isolates from food of animal origin [69]. Similarly, the presence of gelatinase encoding gene (*gelE*) was also frequently demonstrated in* E. faecalis *clinical and meat isolates, while it was rarely seen in clinical* E. faecium *isolates (Table 2). This corresponds with previous reports on high proportions of* E. faecalis *and low proportions of* E. faecium* isolates from meat harboring* gelE* [42, 71, 72].

### 3.3. Association between Antimicrobial Resistance and Virulence Genes

In* E. faecalis*, a moderate positive correlation between the phenotypic resistance and virulence genes was observed (Spearman correlation coefficient* r*_*S*_=51.4%,* p*<0.0001). Similarly, in* E. faecium*, a strong positive correlation was observed (*r*_*S*_=71.9%,* p*<0.0001). Several significant associations between the antimicrobial resistance phenotype and virulence genes were identified (Table S3 and Table S4). In* E. faecalis*, the presence of* asa1*,* esp*, and* cylA* genes was significantly associated with the resistance to ERY, GEN, and TET (Table S3). In* E. faecium*, the presence of* esp *and* hyl* genes was significantly associated with the phenotypic resistance to AMP, CIP, ERY, GEN, and TGC (Table S4). In both species, a positive association between the presence of virulence genes and phenotypic resistance was more evident in clinical isolates in comparison with meat isolates. This suggests there is a cooccurrence of resistance and virulence determinants in clinical isolates belonging to both analyzed species, possibly due to the antimicrobial treatment favoring the coselection of both traits.

## 4. Conclusions

Herein, we revealed a relatively favorable situation regarding the resistance of* E. faecalis* and* E. faecium* isolates from human clinical specimens and red meat as all isolates were susceptible to daptomycin, linezolid, teicoplanin, and vancomycin, which is of particular importance since these agents are categorized as CIA for human infections. In addition, a considerably higher proportion of susceptible isolates from meat compared to clinical isolates was shown. Only 1.7% of meat isolates were MDR compared to 42.6% of clinical isolates. Therefore,* E. faecalis* and* E. faecium* from red meat most likely do not represent an important source of resistant strains for human colonization of infection. Clinical* E. faecalis* isolates showed an increased presence of virulence genes as 47.9% of isolates harbored more than five virulence genes simultaneously compared to 5% of meat* E. faecalis* isolates. However, the most common combinations of virulence genes in* E. faecalis* isolates from beef and pork, including* efaA*,* ace*, and* gelE*, revealed a similarity in virulence characteristics to human isolates. Even though the most frequent virulence gene patterns in the red meat isolates were less common in human isolates, beef and pork could be regarded as a source of virulent* E. faecalis *strains. In contrast, the red meat could not be assumed as an important vehicle for the transmission of virulent* E. faecium.*

## Figures and Tables

**Table 1:** Overview of the susceptibility testing results for 191 *Enterococcus faecalis* and 51 *Enterococcus faecium* isolates from human clinical specimens and red meat.

Resistance to no. of antimicrobials	Human clinical specimens No. of isolates [%]		Red meat No. of isolates [%]	
	*E. faecalis* (*n*=71)	*E. faecium* (*n*=30)	*E. faecalis* (*n*=120)	*E. faecium* (*n*=21)
6	1 [1.4]	3 [10.0]	0 [0.0]	0 [0.0]
5	4 [5.6]	^*∗∗∗*^13 [43.3]	0 [0.0]	0 [0.0]
4	4 [5.6]	3 [10.0]	0 [0.0]	0 [0.0]
3	^*∗∗*^12 [16.9]	3 [10.0]	2 [1.7]	0 [0.0]
**MDR**	^*∗∗∗∗*^21****[29.6]	^*∗∗∗∗*^**22 **[73.3]	**2 **[1.7]	**0 **[0.0]
2	^*∗∗∗*^14 [19.7]	3 [10.0]	4 [3.3]	1 [4.8]
1	27 [38.0]	2 [6.7]	39 [32.5]	2 [9.5]
Susceptible	9 [12.7]	3 [10.0]	^*∗∗∗∗*^75 [62.5]	^*∗∗∗∗*^18 [85.7]

Total	101		141	

Note: significant associations (the Fisher's exact test) of antimicrobial resistance/susceptibility and origin of isolation for each species are indicated with asterisks. Significance levels: ^*∗∗*^*p*<0.005; ^*∗∗∗*^*p*<0.0005, ^*∗∗∗∗*^*p*<0.0001.

MDR denotes the multidrug resistance.

**Table 2:** The presence of virulence genes in 191 *Enterococcus faecalis* and 51 *Enterococcus faecium *isolates originating from human clinical specimens and red meat.

Virulence gene	Human clinical specimens No. of isolates [%]		Red meat No. of isolates [%]	
	*E. faecalis*	*E. faecium*	*E. faecalis*	*E. faecium*
*ace*	54 [76.1]	4 [13.3]	92 [76.7]	0 [0.0]
*asa1*	^*∗∗∗∗*^46 [64.8]	2 [6.7]	38 [31.7]	0 [0.0]
*cylA*	^*∗∗∗∗*^32 [45.1]	2 [6.7]	6 [5.0]	0 [0.0]
*efaA*	69 [97.2]	4 [13.3]	115 [95.8]	0 [0.0]
*esp*	^*∗∗∗∗*^51 [71.8]	^*∗∗∗∗*^21[70.0]	13 [10.8]	0 [0.0]
*gelE*	^*∗∗∗*^63 [88.7]	2 [6.7]	79 [65.8]	0 [0.0]
*hyl*	2 [2.8]	^*∗∗∗∗*^16 [53.3]	0 [0.0]	0 [0.0]

Total	71	30	120	21

*ace*: collagen‐binding protein;* asa1*: aggregation substance;* cylA*: cytolysin; *efaA*: endocarditis antigen; *esp*: enterococcal surface protein; *gelE*: gelatinase; *hyl*: hyaluronidase.

Note: significant associations (the Fisher's exact test) of the presence of virulence gene and origin of isolation for each species are indicated with asterisks. Significance levels: ^*∗∗∗*^*p*<0.0005; ^*∗∗∗∗*^*p*<0.0001.

**Table 3:** Virulence gene patterns observed in *Enterococcus faecalis* and *Enterococcus faecium* isolates from human clinical specimens and red meat.

Virulence gene pattern	Human clinical specimens No. of isolates		Red meat No. of isolates	
	*E. faecalis*	*E. faecium*	*E. faecalis*	*E. faecium*
*ace-asa1-cylA-efaA-esp-gelE*	22	1	0	0
*ace-asa1-cylA-efaA-gelE*	2	0	1	0
*ace-asa1-efaA-esp-gelE*	2	0	0	0
*ace-efaA-asa1-esp-cylA*	4	0	4	0
*asa1-cylA-efaA-esp-gelE*	4	0	1	0
*ace-asa1-cylA-efaA*	0	1	0	0
*ace-asa1-efaA-gelE*	9	0	17	0
*ace-efaA-esp-gelE*	7	1	3	0
*asa1-efaA-esp-gelE*	2	0	1	0
*ace-asa1-efaA*	0	0	9	0
*ace-efaA-esp*	1	1	0	0
*ace-efaA-gelE*	7	0	39	0
*asa1-efaA-esp*	1	0	0	0
*asa1-efaA-gelE*	0	0	3	0
*efaA-esp-gelE*	6	0	4	0
*ace-efaA*	0	0	19	0
*asa1-efaA*	0	0	1	0
*asa1-gelE*	0	0	1	0
*efaA-gelE*	2	0	8	0
*esp-hyl*	2	16	0	0
*efaA*	0	0	5	0
*esp*	0	2	0	0
*gelE*	0	0	1	0
None	0	8	3	21

Total	71	30	120	21

## Data Availability

The data used to support the findings of this study are included within the article and the supplementary information file. The raw data on isolation source, antibiotic susceptibility, and presence of virulence genes for each isolate are available from the corresponding author upon request.

## Supplementary Materials

Supplementary Materials Table S1.* Distribution of MICs and resistance [*%*] in E. faecalis (n=71) and E. faecium (n=30) from human clinical specimens*. Data on MIC [*μ*g/ml] distribution, including MIC_50_ and MIC_90_, and resistance [%] to the tested antimicrobials of* E. faecalis* and* E. faecium* isolates from human clinical cases is shown. Table S2.* Distribution of MICs and resistance [*%*] in E. faecalis (n=120) and E. faecium (n=21) from red meat*. Data on MIC [*μ*g/ml] distribution, including MIC_50_ and MIC_90_, and resistance [%] to the tested antimicrobials of* E. faecalis* and* E. faecium* isolates from beef and pork is shown. Table S3.* Association between phenotypic antimicrobial resistance and virulence genes for E. faecalis isolates from human clinical specimens and red meat (n=191)*. The numbers denote* p* values obtained using the Fisher's exact test; only groups with an expected frequency of >5 were compared. Table S4.* Association between phenotypic antimicrobial resistance and virulence genes for E. faecium isolates from human clinical specimens and red meat (n=51)*. The numbers denote* p* values obtained using the Fisher's exact test; only groups with an expected frequency of >5 were compared.
