# Supplementary material for: Antimicrobial Resistance and Virulence Genes in Enterococcus faecium and Enterococcus faecalis from Humans and Retail Red Meat
Source: Biomed Res Int. 2019 May 9;2019:2815279. doi: 10.1155/2019/2815279 (PMC6532320; doi:10.1155/2019/2815279)
Supplement: Supplementary Materials — Table S1. Distribution of MICs and resistance [%] in E. faecalis (n=71) and E. faecium (n=30) from human clinical specimens. Data on MIC [μg/ml] distribution, including MIC50 and MIC90, and resistance [%] to the tested antimicrobials of E. faecalis and E. faecium isolates from human clinical cases is shown. Table S2. Distribution of MICs and resistance [%] in E. faecalis (n=120) and E. faecium (n=21) from red meat. Data on MIC [μg/ml] distribution, including MIC50 and MIC90, and resistance [%] to the tested antimicrobials of E. faecalis and E. faecium isolates from beef and pork is shown. Table S3. Association between phenotypic antimicrobial resistance and virulence genes for E. faecalis isolates from human clinical specimens and red meat (n=191). The numbers denote p values obtained using the Fisher's exact test; only groups with an expected frequency of >5 were compared. Table S4. Association between phenotypic antimicrobial resistance and virulence genes for E. faecium isolates from human clinical specimens and red meat (n=51). The numbers denote p values obtained using the Fisher's exact test; only groups with an expected frequency of >5 were compared. [file 2815279.f1.pdf]

Table S1: Distribution of MICs and resistance [%] in *E. faecalis* (*n*=71) and *E. faecium* (*n*=30) from human clinical specimens.

Antimicrobial agent	Resistance [%]	MIC [µg/ml]																		Range	MIC_{50}	MIC_{90}
E. faecalis (*n*=71)		0.016	0.03	0.06	0.12	0.25	0.5	1	2	4	8	16	32	64	128	256	512	1024	2048			
Ampicillin (AMP)	2.8					21		41	12	1					2					≤0.5->64	1	2
Chloramphenicol (CHL)	5.6								28		39			3	1					≤4-128	8	8
Ciprofloxacin (CIP)	18.3					1	8	32	17			1	12							0.25->16	1	>16
Daptomycin (DAP)	0				5		7	30	28	1										≤0.25-4	1	2
Erythromycin (ERY)	46.5						14		12	12	1	1	2			29				≤1->128	4	>128
Gentamicin (GEN)	28.2									33		16	2			2		6	12	≤8->1204	16	>1024
Linezolid (LZD)	0					3		23	44	1										≤0.5-4	2	2
Teicoplanin (TEI)	0					71														≤0.5	≤0.5	≤0.5
Tetracycline (TET)	78.9						15						5	45	6					≤1-128	64	64
Tigecycline (TGC)	7				26	40	5													0.12-0.5	0.25	0.25
Vancomycin (VAN)	0						44		19	8										≤1-4	≤1	4

Antimicrobial agent	Resistance [%]	MIC [µg/ml]																		Range	MIC_{50}	MIC_{90}
E. faecium (*n*=30)		0.016	0.03	0.06	0.12	0.25	0.5	1	2	4	8	16	32	64	128	256	512	1024	2048			
Ampicillin (AMP)	70.0							4	4	1	1				20					1->64	>64	>64
Chloramphenicol (CHL)	0								15		14		1							≤4-32	≤4	8
Ciprofloxacin (CIP)	70.0							4		5			21							1->16	>16	>16
Daptomycin (DAP)	0						1	15	10	4										0.5-4	1	4
Erythromycin (ERY)	76.7						1		2	4	1	1				21				≤1->128	>128	>128
Gentamicin (GEN)	56.7									13						1			16	≤8->1204	>1204	>1204
Linezolid (LZD)	0							17	13											1-2	1	2
Quinupristin/dalfopristin (SYN)	56.7					2		6		5	15	2								≤0.5-16	8	8
Teicoplanin (TEI)	0					29		1												≤0.5-1	≤0.5	≤0.5
Tetracycline (TET)	13.3						26							3	1					≤1-128	≤1	64
Tigecycline (TGC)	30.0				13	8	8	1												0.12-1	0.25	0.5
Vancomycin (VAN)	0						27		3											≤1-2	≤1	≤1

Note: Numbers in the tables are the numbers of isolates with the corresponding MIC. White area indicates the range of dilutions tested for each antimicrobial agent. Values under this range denote MICs at lower or equal than the lowest concentration tested, and values above this range denote MICs greater than the highest concentration tested. Vertical lines indicate epidemiological cut-offs (ECOFFs) according to the EUCAST guidelines.

Table S2: Distribution of MICs and resistance [%] in *E. faecalis* (*n*=120) and *E. faecium* (*n*=21) from red meat.

Antimicrobial agent	Resistance [%]	MIC [µg/ml]																		Range	MIC_{50}	MIC_{90}
E. faecalis (*n*=120)		0.016	0.03	0.06	0.12	0.25	0.5	1	2	4	8	16	32	64	128	256	512	1024	2048			
Ampicillin (AMP)	0					27		79	14											≤0.5-2	1	2
Chloramphenicol (CHL)	0								73		47									≤4-8	≤4	8
Ciprofloxacin (CIP)	0.8			1		1	25	75	17		1									≤0.12-8	1	2
Daptomycin (DAP)	0				5		15	59	39	2										≤0.25-4	1	2
Erythromycin (ERY)	0						48		55	13			1			3				≤1->128	2	4
Gentamicin (GEN)	1.7									83		33	2						2	≤8->1204	≤8	16
Linezolid (LZD)	0					6		28	86											≤0.5-2	2	2
Teicoplanin (TEI)	0					120														≤0.5	≤0.5	≤0.5
Tetracycline (TET)	29.2						84		1				2	21	11	1				≤1->128	≤1	64
Tigecycline (TGC)	9.2	9		24	57	19	11													≤0.03-0.5	0.12	0.25
Vancomycin (VAN)	0						81		39											≤1-2	≤1	2
E. faecium (*n*=21)		0.016	0.03	0.06	0.12	0.25	0.5	1	2	4	8	16	32	64	128	256	512	1024	2048			
Ampicillin (AMP)	0					8		9	3	1										≤0.5-4	1	2
Chloramphenicol (CHL)	0								15		5		1							≤4-32	≤4	8
Ciprofloxacin (CIP)	4.8			2		1		5	6	6	1									≤0.12-8	2	4
Daptomycin (DAP)	0				1		2	2	8	8										≤0.25-4	2	4
Erythromycin (ERY)	4.8						5		12	3						1				≤1->128	2	4
Gentamicin (GEN)	0									20		1								≤8-16	≤8	≤8
Linezolid (LZD)	0							5	16											1-2	2	2
Quinupristin/dalfopristin (SYN)	4.8					6		4	2	8	1									≤0.5-8	2	4
Teicoplanin (TEI)	0					21														≤0.5	≤0.5	≤0.5
Tetracycline (TET)	4.8						20									1				≤1->128	≤1	≤1
Tigecycline (TGC)	0	4		5	11	1														≤0.03-0.25	0.12	0.12
Vancomycin (VAN)	0						19		1	1										≤1-4	≤1	≤1

Note: Numbers in the tables are the numbers of isolates with the corresponding MIC. White area indicates the range of dilutions tested for each antimicrobial agent. Values under this range denote MICs at lower or equal than the lowest concentration tested, and values above this range denote MICs greater than the highest concentration tested. Vertical lines indicate epidemiological cut-offs (ECOFFs) according to the EUCAST guidelines.

Table S3: Association between phenotypic antimicrobial resistance and virulence genes for *E. faecalis* isolates from human clinical specimens and red meat (*n*=191).

Antimicrobial	Virulence genes					
	ace	*asa1*	*cylA*	*efaA*	*esp*	*gelE*
Ciprofloxacin (CIP)	0.5258	**0.0478**	0.3091	0.4183	0.5574	0.5250
Erythromycin (ERY)	0.5243	**<0.0001**	**<0.0001**	0.6227	**<0.0001**	0.3004
Gentamicin (GEN)	0.7894	**0.0002**	**0.0190**	0.1863	**0.0001**	0.2975
Tetracycline (TET)	0.1281	**<0.0001**	**<0.0001**	0.4481	**<0.0001**	**0.0026**
Tigecycline (TGC)	1.0000	0.1235	0.7431	**0.0139**	1.0000	0.3680

Note: The numbers denote *p* values obtained using the Fisher's exact test; only groups with an expected frequency of >5 were compared. Significant *p* values ($p<0.05$) are shown in bold; *p* values <0.0005 indicate strong association.

Table S4: Association between phenotypic antimicrobial resistance and virulence genes for *E. faecium* isolates from human clinical specimens and red meat (*n*=51).

Antimicrobial	Virulence genes	
	esp	*hyl*
Ampicillin (AMP)	**<0.0001**	**<0.0001**
Ciprofloxacin (CIP)	**<0.0001**	**<0.0001**
Erythromycin (ERY)	**<0.0001**	**0.0002**
Gentamicin (GEN)	**0.0006**	**<0.0001**
Tigecycline (TGC)	**<0.0001**	**0.0002**

Note: The numbers denote *p* values obtained using the Fisher's exact test; only groups with an expected frequency of >5 were compared. Significant *p* values ($p<0.05$) are shown in bold; all values indicate strong association ($p<0.0005$).
